# Co-creating support for adolescents with long-lasting pain: findings from workshops with adolescents, parents, and professionals

**DOI:** 10.1186/s12913-025-13654-0

**Published:** 2025-11-24

**Authors:** Maren Hjelle Guddal, Simon Kristoffer Johansen, Kirsti Riiser, Turid Sundar, Trygve Skonnord, Michael S. Rathleff, Kate M. Dunn, Kaja Smedbråten, Britt Elin ∅iestad, Henriette Jahre

**Affiliations:** 1 Department of Rehabilitation Science and Health Technology and Centre for Intelligent Musculoskeletal Health (CIM), Faculty of Health Sciences, OsloMet, Oslo, Norway; 2 Center for General Practice (CAM-AAU), Aalborg University, Aalborg, Denmark; 3 Department of Health Science and Technology, Faculty of Medicine, Aalborg University, Aalborg, Denmark; 4 Child and Adolescent Health Promotion Services, Norwegian Institute of Public Health, Levanger, Norway; 5 Department of Nursing and Health Promotion, Faculty of Health Sciences, OsloMet, Oslo, Norway; 6 Department of General Practice, Institute of Health and Society, University of Oslo, Oslo, Norway; 7 Centre for Musculoskeletal Health Research, School of Medicine, Keele University, Keele, UK

**Keywords:** Adolescent long-lasting pain, Person-centered intervention, Participatory research, Co-design, School-health services, Interdisciplinary collaboration

## Abstract

**Introduction:**

Long-lasting pain in adolescents may affect education, social interactions, and is associated with mental health challenges. Current interventions are often suboptimal due to insufficient understanding of the challenges faced by adolescents with long-lasting pain and those who support them. Understanding the management challenges experienced by adolescents with long-lasting pain, along with those faced by their parents, education professionals, and healthcare professionals (HCPs), is crucial for informing person-centered interventions and improving care and outcomes.

**Objectives:**

This study aimed to gather insights and visions from adolescents with long-lasting pain, along with their parents, HCPs, and teachers, to develop a person-centered intervention for managing pain.

**Methods:**

We used a qualitative Action Research approach, employing three workshops with 1) adolescents with long-lasting pain, 2) HCPs and teachers, and 3) parents. Workshops incorporated case vignettes and design-card exercises to foster dialogue, knowledge construction and articulation of insights and visions to inform intervention design. Data were collected through audio recordings, participant artifacts, and field notes, then analyzed using Reflexive Thematic Analysis and matrix synthesis to create a conceptual model highlighting tension points for future interventions.

**Results:**

In three separate workshops, 13 adolescents with long-lasting pain, 16 HCPs and teachers (four physiotherapists, four senior high school teachers, three psychologists, three school health nurses, and two General Practitioners), and four parents participated. Adolescents described pain’s pervasive impact on their education, social lives, and self-identity. Barriers to improvement included limited coordination between healthcare and school, as well as a lack of communication. The school setting and school health services were identified as an ideal setting for interventions. Key visions for interventions included early holistic assessments, enhanced interdisciplinary collaboration with dedicated coordination roles, specialized adolescent-focused expertise, and the use of digital tools for personalized management.

**Conclusion:**

This study brought new insights into the development of a person-centered intervention for adolescents with long-lasting pain, highlighting the impact of pain on those affected and barriers to optimal care. It emphasized the need for including education professionals and school health services in interdisciplinary collaboration, holistic assessments, and improved expertise in adolescent pain management.

**Supplementary information:**

The online version contains supplementary material available at 10.1186/s12913-025-13654-0.

## Introduction

Long-lasting pain in adolescents (age 10 to 19) is complex and common with possible far-reaching consequences. A considerable proportion (20–44%) of adolescents experience pain persisting beyond 3 months, with prevalence increasing with age, being higher among girls, and influenced by socio-cultural factors such as economic status and family background [1–4]. Adolescents with long-lasting pain face multiple challenges, including high consumption of analgesics, school absenteeism, disability, and psychological distress [4–11]. Furthermore, long-lasting pain has been shown to be strongly associated with reduced health-related quality of life (HQOL) in adolescents [12, 13], impeded psycho-social development, reduced educational and vocational prospects [14–17], and increases in risk of chronic pain and pain-related comorbidities during adulthood [7, 18].

Despite the profound impact of long-lasting pain, there remains a lack of evidence-based guidelines and preventative measures to address the condition in adolescents. Interventions that encompass self-management education [19, 20], psychological interventions [21–24], and various exercise modalities [20, 25] have shown potential for improving outcomes. Ensuring that adolescents acquire the knowledge, skills, and confidence to autonomously self-manage their conditions in everyday settings is pivotal for improving short- and long-term outcomes [13, 26, 27].

While adolescents with long-lasting pain call for treatments tailored to their individual needs [28], this effort is hindered by a lack of knowledge about the everyday management challenges and barriers they face, and the contexts in which these occur. [28, 29]. Patient education literature describes adolescents as challenging to support due to the transitional nature of adolescence [30], their ‘right now’ view on illness, and their desire for independence from parents [31, 32]. Qualitative studies describe how adolescents progressively acquire management competencies by overcoming challenges [33–35]. Studies also illustrate how adolescents entering treatment face additional management tasks, such as monitoring symptoms, managing medications, making decisions, using tools, and communicating pain to parents or healthcare professionals (HCPs) to successfully comply with treatments [36, 37]. Furthermore, research on health literacy implies that adolescents’ management skills are distributed, as they rely on the competencies of parents, peers, and HCPs to support health decision-making in clinical and everyday settings [38–41]. Incorporating perspectives from adolescents and those supporting them, such as HCPs, teachers, and parents, is essential for understanding the complex interplay between adolescents’ management challenges, contextual determinants, and the roles these stakeholders play in the management of pain [42, 43]. This comprehensive approach is innovative and often overlooked in previous research, and it could provide valuable insights for designing novel treatment concepts.

By utilizing a participatory approach, this study aims to collect insights from adolescents with long-lasting pain, as well as from HCPs, parents, and teachers. It seeks to understand the challenges and barriers they face and to gather their visions for developing a person-centered intervention that supports adolescents in managing long-lasting pain. Addressing a gap in the literature, this study explores how pain management is influenced by interactions across different contexts and stakeholders, offering a more holistic understanding of how to support adolescents in their daily lives.

## Methods

### Study design

This qualitative study was conceptualized using Action Research as a methodological framework to guide our application of methods, data analysis and presentation of findings [44, 45]. The intervention segment comprised three workshops, using the future workshop approach [46, 47], one with adolescents with long-lasting pain, one with healthcare- and education professionals and one with parents of adolescents with long-lasting pain. All workshops followed the same procedure, utilizing case vignettes and inspiration cards to stimulate dialogue and shared knowledge construction [48–50]. Participant discussions were captured via audio recorders, flipboards and photography, transcribed and analyzed using reflective thematic analysis [51]. The insights and visions from each analysis were synthesized though a matrix analysis [52] to map challenges, interactions, tensions and visions as design opportunities [53]. The study was reported in accordance with the Consolidated Criteria for Reporting Qualitative Research (COREQ) checklist [54] to ensure quality and transparency in our communication of this study.

### Ethics

Our study protocol was registered with the Open Science Framework prior to data collection on November 24, 2023 [55]. The study was assessed by the Regional Committees for Medical and Health Research Ethics (REK, ID 607139), which determined that ethical approval was not required. Additionally, The Norwegian Agency for Shared Services in Education and Research (SIKT) reviewed and confirmed the legality of the personal data processing (ref. 324868). Based on their decision, written consent from participants was not required. All participants were provided with both written and oral information about data management and processing. They gave verbal consent after confirming they had read and understood the terms of participation.

### Research group

The research team consisted of two postdoctoral researchers (MHG, HJ), both female physiotherapists holding PhDs, one female Professor (BEØ) with a background in physiotherapy, all with moderate experience in qualitative methods, and one male PhD student (SKJ) with a background in Information Science and extensive knowledge of qualitative and future workshop methods. Two female researchers, one Professor (KR) and one PhD (TBS), with expertise in qualitative research, assisted with the data analysis. Finally, the research group included two other professors (MR, KD), one male general practitioner (TS), and one female PhD (KS) with a background in physiotherapy, who provided ongoing sparring and aided in manuscript preparation.

### Recruitment procedure

Study population 1 (SP1) consisted of adolescents aged 16–19 years who had experienced pain lasting for 3 months or more, aligned with the current definition of chronic pain supported by the International Association for the Study of Pain (IASP) [56]. We recruited through posters, social media, information dissemination at school nurses’ offices, and direct outreach to adolescents via their digital school-platforms. Information about the project with a link to a registration form was provided. Study population 2 (SP2) consisted of HCPs (physiotherapists, school health nurses, general practitioners (GPs), psychologists) and teachers at senior high schools. Recruitment of HCPs was conducted through requests from the research group’s network of practitioners. Recruitment of teachers was conducted through requests to schools in the Oslo area. The third study population (SP3) consisted of parents of adolescents with pain for at least 3 months, recruited independently of those in the workshop for adolescents. We utilized posters and social media for recruitment, along with a link to a registration form.

### Future workshops

**Fig. 1:**
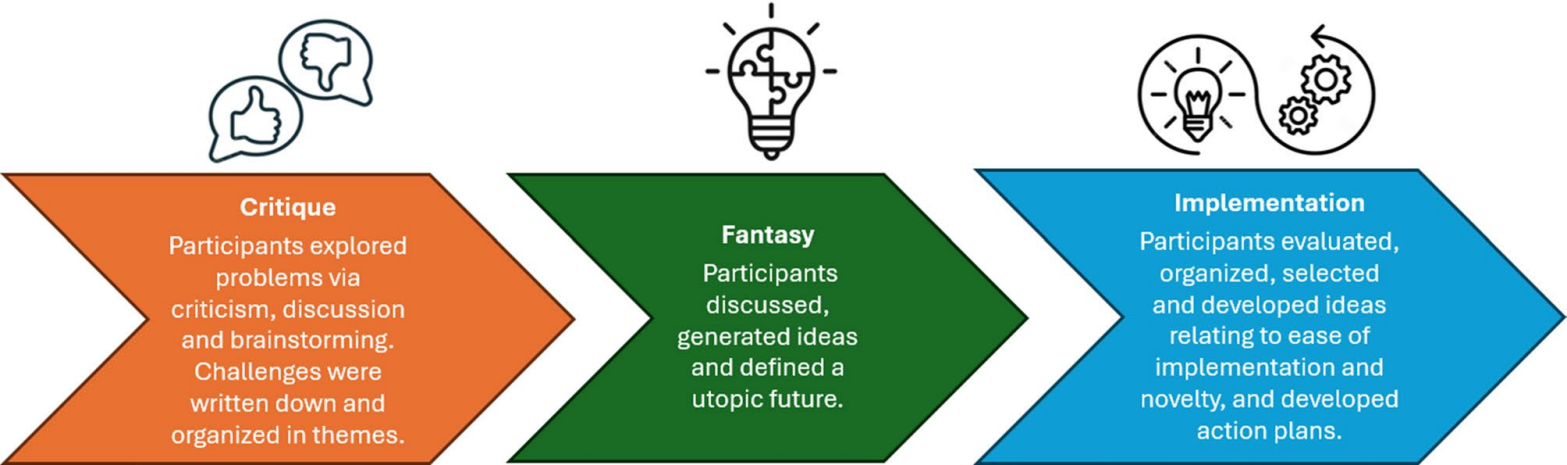
Overview of the future workshop phases. *The phase descriptions were adopted from Vidal et al.* [47] *and* *Johansen et al.* [40]. *A follow-up phase is part of the method but not described in this article. This phase involves initiating, monitoring, and adjusting action plans, and planning new workshops to address implementation challenges*

The Future workshop method as described by Apel [46] and Vidal et al. [47] informed the design of the workshops. The method was selected from its focus on leveraging dialogue, generative activities, and the co-construction of knowledge to define novel solutions to complex real-world problems [46, 47]. This is archived via a three-step process involving critique, idea generation and discussion implementation to facilitate participants’ articulation of one or more shared visions of desirable futures and action plans for achieving these changes [57] (Fig. 1).

The decision to conduct three separate workshops (SP 1, 2 & 3) was made to reduce the potentials for asymmetric power dynamics, and to create a safe environment where participants could share their personal experiences without fear of judgement [58]. A generative exercise, which utilized three case vignettes [48] and inspiration card games [49] was developed to foster discussion and provide guidance throughout all phases of the workshops. The initial content for the case vignettes and inspiration cards was proposed by the researchers and revised based on feedback from the extended research team. The workshop activities were pilot tested for comprehension by testing the content of all phases, including case vignettes and inspiration cards, with two adolescents, a physiotherapist and researcher, and a parent of an adolescent with persistent pain.

#### Case vignettes

Case vignettes were used as a foundation for the workshop activities (SP 1–3), helping to initiate discussions and card exercises [48]. Three different imaginary cases were developed to illustrate different challenges and support needs faced by adolescents with long-lasting pain. One case described a stressed and overloaded 16-year-old girl with neck pain and headaches, concerned about her future. Another case involved a 17-year-old boy facing shoulder pain and contemplating high school dropout. The third case described a 16-year-old girl from a minority background experiencing widespread pain while struggling to manage her weight and exercise due to pain (Appendix 1).

#### Design cards

To guide participant interactions, foster and promote dialogue, co-creation, and to assist our participants in bridging the gaps between the three workshop phases, each workshop utilized inspiration cards [49]. These cards supported shifts in focus during the workshop phases and were used to create a safe environment for individuals to actively participate in the process and contribute to the discussion [49].

The inspiration cards were divided into three categories connected to conceptual domains: Domain cards, Problem cards, and Solution cards. The Domain cards (blue) illustrated where adolescents experience pain, such as at school, at home, or during leisure time, and were introduced during the Critique phase to help participants identify and discuss relevant issues. Problem cards (black) represented specific challenges or barriers associated with living with long-lasting pain and were also distributed during the Critique phase, enabling participants to articulate and explore problems. Participants could combine these cards by linking specific challenges to domains (e.g., discussing problems associated with school or at home). Solution cards (green) were introduced in the Fantasy phase and included suggestions or prompts for possible interventions or solutions. Participants were encouraged to add these Solution cards to the Domain and Problem card combinations they had created, facilitating structured brainstorming and the imagining of ways to address the identified challenges (Appendix 2). In addition to the cards and case vignettes, three distinct sets of sticky notes in red, yellow, and green were used. Participants had pens and markers to write notes and create additional design cards when new topics evolved during dialogues and discussions.

### Settings and procedure

The workshops were held within the locality of the Faculty of Health Sciences at Oslo Metropolitan University between the dates of 29.01.24 and 12.02.24. The three workshops followed similar procedures, lasted for 3 hours and included three 40-minute work phases and breaks [46, 47]. Each workshop was conducted with a facilitator (HJ) who led the workshop activities and two to three coordinators (MHG, BEØ, SKJ) who monitored workshop activities, kept track of time, co-facilitated and handled practical tasks. The workshops were initiated with a 25 min presentation, where one coordinator (MHG) introduced the project, the research group, the reasons for conducting the research, the future workshop method and workshop activities. The facilitator (HJ) then divided participants into workgroups of three to five participants, before introducing the case vignettes, inspiration cards and providing exercise instructions.

During each workshop phase, the facilitator and coordinators monitored the participants’ work from the background and stepped in to answer questions or avoid group discussions stagnated. After each work-phase, participants met in the main room for plenary discussions, where they presented their thoughts, ideas and visions while the facilitator summarized key points on a flip board and asked follow-up questions. This process was repeated for the Critique, Fantasy and Vision phase. Upon completion of phase three, each group presented their visions for a treatment approach for adolescents with long-lasting pain and received feedback from the other groups. Each workshop ended with a debriefing where the facilitator and a coordinator (HJ, MHG) informed participants about their rights and answered questions.

### Data collection

The discussions from the workshops were captured through audio recordings, flipboards and photography of sticky notes and card games. This included both the discussions in the main room during transitions between phases and the notes and outputs generated within each workgroup. The audio recordings were conducted through a voice recorder application (nettskjema.no) [59] with directly transfer to a secure storage infrastructure, the TSD (Tjenester for Sensitive Data), owned by the University of Oslo, operated and developed by the TSD service group at the University of Oslo. Photo documentation of the insights and visions noted on the flipboards, sticky notes and inspiration cards were uploaded to a secure server, to be included to support the data analysis.

### Data analysis

Audio recordings were transcribed using the University of Oslo’s internal service Autotext (Whisper speech recognition system), then manually checked by a researcher (MHG or HJ) to ensure meaning retention [60] and organized to be interpreted via separate analysis. The transcribed workshop data was analyzed separately by three members of the research group (MHG, HJ, SKJ) via the reflective thematic analysis approach by Braun and Clarke [51] using NVivo version 1.7 coding software. The analytical process was conducted as a six-step process which included 1) familiarization, 2) coding, 3) identifying preliminary themes, 4) reviewing and theme development, 5) refinement and 6) synthesis into storybook themes via matrix analysis. During the analysis, each researcher (MHG, HJ, SKJ) was responsible for analyzing the transcripts from one of the three workshops. The coding was reviewed by two external researchers (KR, TBS) to ensure coding integrity, and that all topics had been included. Themes and subthemes for each workshop were identified by merging codes, condensed and refined to reduce thematic overlaps (Appendix 3). Throughout the refinement and synthesis process the researchers (MHG, HJ, SKJ) presented and discussed the identified themes and subthemes between themselves, before presenting them to KR and TBS for feedback, to avoid tunneling and ensure the essence of the data had been captured. Finally, the identified themes from all three workshops were synthesized via a matrix analysis [52]. This approach involved organizing the data into a matrix to explore how domains (e.g., healthcare, education, family), actors (e.g., adolescents, parents, HCPs, teachers), and their interactions revealed key tensions and barriers within the data. The matrix analysis complemented the thematic analysis by providing a structure to map connections and overlaps between themes, which informed the development of a conceptual model. The final story book themes were written up and presented by MHG, HJ, and SKJ (Appendix 4).

## Results

**Fig. 2:**
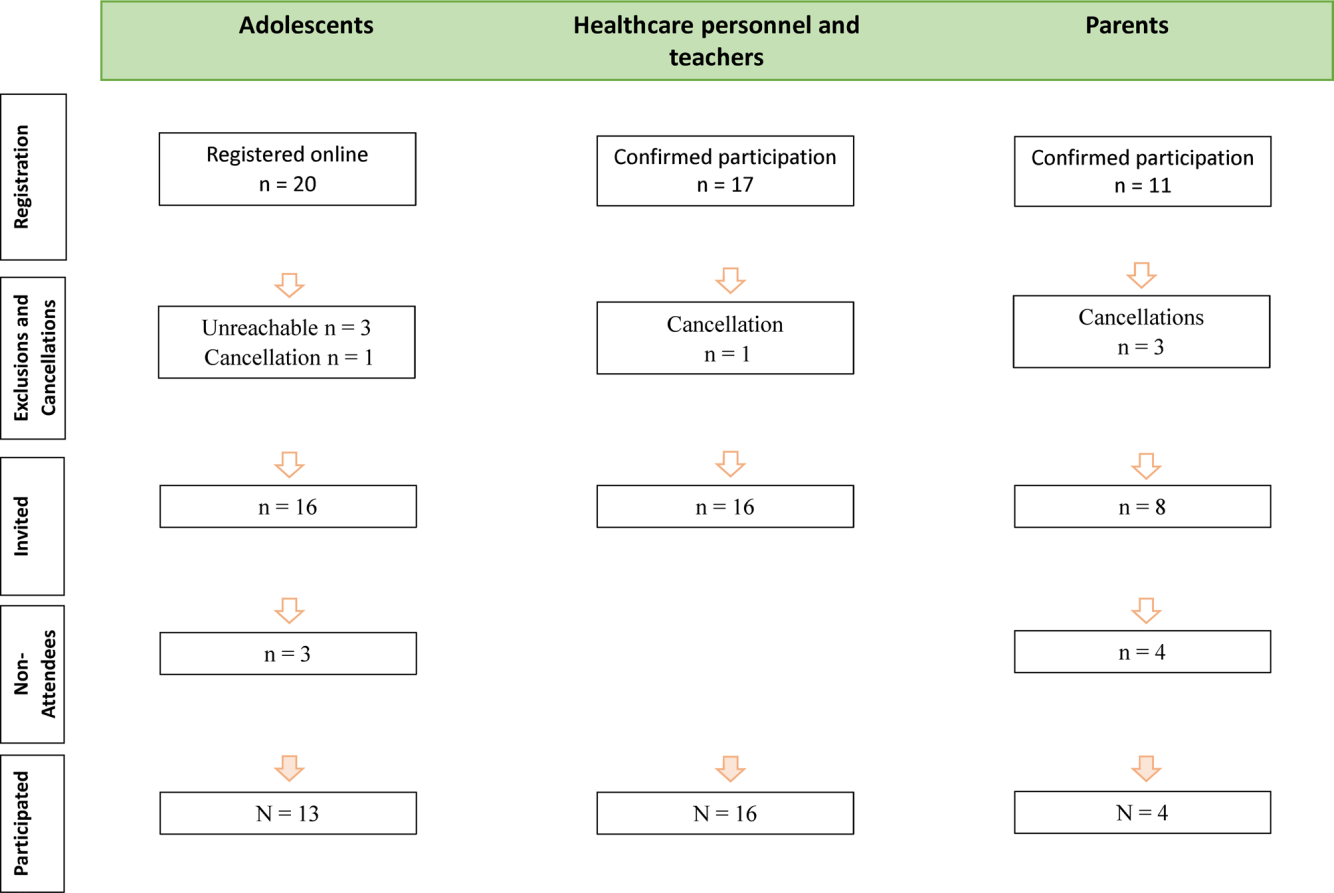
Flow chart inclusion

Out of 20 adolescents who registered as interested, 13 participated in the workshop. For HCPs and teachers, 16 out of 17 confirmed participants attended. Among 11 interested parents, 4 attended the workshop (Fig. 2).

The ages of adolescents ranged from 16 to 19 years. Among them, 12 reported musculoskeletal pain, six reported headaches and one reported other types of pain. Of these, two participants reported pain in only one site, four participants reported pain in two sites, and seven participants reported pain in three or more sites. All had sought medical help, nine used pain medication, eight reported receiving a diagnosis, and nine reported receiving mental health support for emotional or psychological challenges (e.g., anxiety or depression). Notably, six participants reported both using pain medication and receiving mental health support. The HCP and teacher group comprised 16 individuals, with an age range of 26 to 53 years. This group included four physiotherapists, four senior high school teachers, three psychologists, three school health nurses, and two GPs, with an average of 14.4 years (SD = 7.4) of professional experience. The parent group consisted of four individuals, with an age range of 39 to 50 years. Their children experienced musculoskeletal pain, including two also reporting headaches. All children of parents had sought medical help, with two receiving a diagnosis and two seeking support for mental health issues (Table 1).

**Table 1:** Descriptive characteristics of participants

Characteristics	
**WS1 with asolescents**	
Age range (years)	16–19
Sex, n (%)	
Female	10 (77)
Male	3 (23)
Pain duration, n (%)	
6–12 m	1 (8)
1–2 y	3 (23)
3–6 y	6 (46)
> 6 y	3 (23)
Pain location*, n (%)	
Headache	6 (46)
Neckpain	8 (62)
Shoulderpain	5 (39)
Upper back pain	5 (39)
Low back pain	6 (46)
Chest pain	2 (15)
Stomach pain	3 (23)
Arm and/or hand pain	4 (31)
Hip and/or thigh pain	4 (31)
Knee pain	4 (31)
Ankle and/or foot pain	2 (15)
Other pain	2 (15)
Number of pain sites, n (%)	
1	2 (15)
2	4 (31)
≥	7 (54)
Pain Intensity (Typically), mean (SD)	7 (1,58)
Use of analgesics, n (%)	
Yes	9 (69)
No	3 (23)
Missing	1 (8)
Sought healthcare, n (%)	
Yes	13 (100)
Diagnosis, n (%)	
Yes	8 (62)
No	5 (39)
Self-report of diagnose, n	
Migraine	1
Scoliosis and migraine	1
Flatfoot	1
Psoriatic arthritis	1
Cartilage loss in the knee	1
Degenerative joints	1
Sought help for mental health issues, n (%)	
Yes	9 (69)
No	4 (31)
Psychological distress (SCL-5)†,mean (SD)	2,7 (0,65)
**WS2 with healthcare personnel and teachers**	
Age range (years)	26–53
Profession, n (%)	
Physiotherapist	4 (25)
General practitioner	2 (13)
School health nurse	3 (19)
Psychologist	3 (19)
Teacher (senior high school)	4 (25)
Professional experience, years, mean (SD)	14,4 (7)
**WS3 with parents**	
Age range (years)	39 - 50
Pain duration (their adolescent), n (%)	
6–12 m	0 (0)
1–2 y	0 (0)
3–6 y	3 (75)
> 6 y	1 (25)
Pain location*, n	
Headache	2
Neckpain	1
Shoulderpain	0
Upper back pain	1
Low back pain	2
Chest pain	0
Stomach pain	0
Arm and/or hand pain	0
Hip and/or thigh pain	2
Knee pain	4
Ankle and/or foot pain	1
Other pain	1
Sought healthcare, n (%)	
Yes	4 (100)
Diagnosis, n (%)	
Yes	2 (50)
No	2 (50)
Self-report of diagnose, n	
Idiopathic scoliosis	1
Congenital foot deformity (clubfoot)	1
Sought help for mental health issues	
Yes	2 (50)
No	2 (50)

* More than 1 could be selected

† SCL-5, Symptom Check List Five item

The reflective thematic analysis identified six main themes outlining the roles of adolescents, HCPs and parents, interactions, systemic and domain-specific barriers, and visions for future person-centered intervention for adolescents with long-lasting pain. We elaborate on each of these below.

### Theme 1: Adolescents as navigators of long-lasting pain, relationships and social situations

Adolescents described their long-lasting pain as complex. This pain occurred in different contexts such as at home, at school, during sports or leisure activities, and it varied in intensity. Several adolescents reported experiencing fear of their condition worsening and concerns about serious illness. However, this diminished as they learned to identify symptoms, recognized pain patterns and managed their personal resources over time. They highlighted the invisibility nature of pain as a major obstacle for managing their condition, as they experienced how looking ‘okay’ on the outside could lead to misunderstandings when expressing their challenges or support needs to parents, teachers and peers.

Adolescent 4: I actually feel the same about school, with teachers and all that. If you have a chronic illness, for example, it’s like you’re not taken entirely seriously. And because they can’t see it, they think everything is fine.

Adolescents expressed managing their pain conditions through everyday trial-and-error while facing mental and social challenges. Their approaches were shaped by their own experiences with self-management and informed by advice from parents, input from peers, and resources such as the internet. This required being mindful of activities, managing resources to avoid flare-ups or mental burn-out, and ensuring enough energy for exercise and other valued after-school activities. They mentioned using several managements strategies, e.g. activity planning, pacing, taking breaks, withdrawing, nutrition management and medication to balance their pain throughout their day. Struggling with pain management and having to decline social activities frustrated them and negatively impacted their confidence. Despite this, many adolescents described having pushed themselves to participate in activities, risking increased pain, to avoid falling behind socially.

Adolescent 1: It’s something you get from being ill a lot. Low self-confidence. Because that’s how it is; you don’t get to hang out with friends, and then you stop getting invited and stuff, because you always say no (…) I mean, when you sort of withdraw and become less social, it’s hard to become more social again.

Furthermore, educational challenges included struggling to concentrate during classes and difficulties in obtaining authorized absences due to their pain conditions. Participants described how managing pain at school, like withdrawing from class or requesting flexible schoolwork, made them vulnerable to stigma and social sanctions if peers, teachers, or HCPs didn’t acknowledge the impact of their pain.

Adolescents highlighted how their pain affected their ability to maintain a social identity like e.g. being a good student, a good friend or being able to make interesting social media posts. Thus, falling grades due to pain could lead them into a negative spiral, where they gradually reduced participation in valued activities. This could subsequently affect their ability to e.g. maintain their grades or sports participation, as repeated attempts and failures could lead to reduced motivation for studying or social engagement.

Adolescent 1: Because if you start with school, the fact is, if you don’t do well at school, you just become demotivated because you are actually an ambitious and clever girl. And if you get a poor grade when you were actually aiming for a five, which you’ve studied all night for, including enduring the pain in your neck, it leads to you becoming demotivated because you received such a low grade.

Adolescents described navigating the expectations of others, both relating to their performance in school, their peer group or how to respond to pain in everyday situations. They highlighted how parental inclusion or forming working relationships with teachers and HCPs could help relieve performance pressures and provide access to support, work reductions and additional care. However, asking for help made them vulnerable to stigma if parents, teachers or peers failed to recognize their pain. Adolescents expressed how having their requests dismissed was frustrating and could lead to doubt on whether e.g. parents or HCPs were able to help them. This limited their motivation for seeking additional support.

### Theme 2: HCPs’ balancing of roles: meeting adolescents’ needs and ensuring constructive dialogue

Several HCPs described managing adolescents with long-lasting pain as a complex task. All HCPs emphasized the need to better integrate psychosocial and somatic perspectives on pain and addressing uncertainties about complex conditions to patients. GPs noted their lack of awareness about the services available from school health personnel and faced the challenge of navigating the dilemma of ruling out serious pathologies while reducing use of unnecessary procedures and diagnostic imaging.

All HCPs expressed concern that time constraints often hindered a thorough examination of the bio-psycho-social factors contributing to adolescents’ pain.

GP: In my ideal world, as a general practitioner, I would have more time when the patient comes to see me. I would try to determine whether there is a serious underlying issue, a specific diagnosis, or nonspecific chronic pain. …, but I wish I had more time to map out the maintaining factors.

School health nurse: But I think that having the opportunity to meet people where they are, regardless of what I think it might be, is important. … And if you don’t believe them, they feel rejected. I believe my goal is for them to dare to come again next time. Having enough time to be met the first time, that I think is important.

HCPs and teachers reiterated how long-lasting pain impacted adolescents’ school performance, reduced participation in activities, and how social withdrawal could be compounded by adolescents feeling different. Among these discussions, some HCPs observed that adolescents appeared motivated and receptive during consultations. However, a common thread across all HCPs was the emphasis on their responsibility to foster a trusting relationship where adolescents felt welcome to voice their challenges and support needs. Yet, time constraints, absence of a clear diagnosis and limited treatment options made establishing this alliance difficult, as HCPs felt they had no solutions to offer patients. Several HCPs still highlighted being honest as important to avoid adolescents feeling dismissed when seeking treatment for their pain.

Physiotherapist: We also need to be honest with the patient about the challenges in this field. When we do that, I believe we gain trust. Instead of promising a ‘quick fix’ that may not work, which could lead to a loss of trust and patients seeking other providers, which we don’t want. So, it’s about having the courage to address these issues and ensuring that everyone on the team is willing to do the same.

HCPs and teachers expressed concerns about how high expectations from family, peers, and the education system can influence adolescents’ experience and management of pain. Thus, HCPs described having to be mindful when wording treatments to make their recommendations acceptable to adolescents, while avoiding adding additional pressures on the adolescents to recover.

Physiotherapist: So, there are some factors maintaining these pains. But they might not be willing to address those, not willing to … I think if a person is an overachiever (“flink pike”) like that, I might not even recommend her to exercise ... It becomes a performance arena. Everything becomes a performance arena.

### Theme 3: Parents as coordinators, supporters of pain management and health system navigation

The parents discussed challenges related to raising an adolescent with long-lasting pain, which involved providing support, assisting with follow-ups, and navigating the health system. Several participants described how losing access to adolescents’ health records at age 16 made it difficult to assist with follow-ups and provide guidance after consultations and tests.

Parent: I find it so difficult to help. And especially when you don’t get any support, because you don’t. The general practitioner is like ‘Well then, we’ll send you for some blood tests’. And then you get those blood test results, or maybe you don’t because she’s 16 and no one sees the blood tests unless you’ve checked off that you want a letter in the mail. And then nothing happens, and no one follows up with you afterwards.

Furthermore, parents reported how they felt they lacked knowledge and understanding of adolescent long-lasting pain and how this was a barrier for providing support. Thus, parents were faced with the challenge of having to educate themselves on adolescent pain in order to support their children.

Parent: Because you get, like, a maximum of 20 minutes with the general practitioner. And I, for one, have not been very impressed with the information you get there. You kind of have to work for it yourself. And Google, search, and dig, and ask. Not everyone does that.

Another aspect was how parents often felt that GPs didn’t possess the necessary expertise to treat adolescents with long-lasting pain, and the struggle to obtain a diagnosis or getting their child’s absenteeism from school approved from GPs. Furthermore, some parents described taking on a coordinating role to ensure their child received adequate support during school hours and engaging with teachers to ensure their child’s needs were met. This and negotiating with the GPs were time consuming, demanding and hard to manage alongside everyday responsibilities.

Parent: I’ve been alone with my son. And I’ve spent an entire school year at the school, against the school’s wishes, to help him through his issues. But you’re not met, you don’t have that person. Because you as a parent have that coordinator role, instead of it being someone else from the outside.

Additionally, parents expressed struggling with discussing pain issues with their teens, who may resist talking or taking advice. Several parents attributed this to adolescents grappling with finding their own identity while still depending on parental help for health issues. Parents found this situation frustrating, highlighting the need for support.

Parent: Just meeting someone who has the same issues would be helpful. As parents, we also get very tired. But we can only speak for ourselves. It is very frustrating, especially if the adolescents don’t want to listen to you.

### Theme 4: Communication and collaborative care challenges

Communication challenges were identified in all workshops, among adolescents, HCPs, teachers, and parents, often creating tensions within these groups and their collaboration. However, overcoming these barriers was viewed as essential for enhancing cooperation and better supporting adolescents in managing their pain.

Adolescents and HCPs explained how adolescents often struggled with finding the right words to portray their conditions and how this caused tensions in consultations with HCPs, or when asking teachers for support. Some adolescents pointed out that the adolescents’ limited understanding of their pain and uncertainty about whether it required treatment contributed to their limited vocabulary. HCPs echoed this experience, emphasizing the importance of creating a supportive and respectful environment which was accommodated to the adolescents’ experience during consultations, to avoid them feeling dismissed.

Physiotherapist 3: I agree with what you said about thorough examination and the need to embrace uncertainty around musculoskeletal issues. It doesn’t necessarily need to have a specific name for the pain. It can be a matter of saying, ‘Okay, you have this situation, and there are some sustaining factors here.’ There isn’t always a clear treatment option based on evidence, and we have to be willing to accept that.

Furthermore, HCPs emphasized the need to be cautious in how they frame pain, noting that presenting it as a purely psychological issue can be counterproductive with some patients and may lead to tensions, as it can be seen as an oversimplification of their problems. Additionally, it was emphasized that young males in male-dominated occupations might struggle more to address the mental aspects of their pain experiences. Consequently, framing pain in a way that aligns with adolescents’ individual needs became an additional barrier, especially considering the gender differences in pain perception and expression.

Both adolescents, parents, and HCPs described how adolescents’ family backgrounds, relationships with parents and their way of responding to the adolescents’ pain, could influence the adolescent’s perception of their pain and shape care-seeking behaviors. While HCPs suggested that pain could be a way to address personal limitations and manage performance pressures, all groups explained how adolescents often inherited their parents’ understanding of chronic pain.

*Psychologist: But it might also be easier to say you have a headache or neck pain rather than that you’re tired or depressed. It can be a form of communication to say that things aren’t going well, in a way. It depends on the family background and what is easier to talk about.*

Finally, adolescents conveyed how parents could play a supportive role in pain management, assisting them in their trial-and-error processes and navigating the healthcare system. However, this support was complicated by adolescents’ desire to meet their parents’ expectations, unwillingness to discuss their pain with parents, the perceived usefulness of their parents’ advice and whether adolescents felt parents respected their autonomy. All groups noted that while parental involvement can be supportive, complications can arise from low health literacy or unrealistic expectations, which could hinder communication processes and shape adolescents’ understanding of their condition.

### Theme 5: Barriers within the system

Across all workshops, participants highlighted the difficulties of navigating the education and health systems, explaining how these barriers could strain collaboration efforts. One recurring theme was how some adolescents struggled to obtain referrals to specialized care. This often ended in frustration and stress when GPs were not able to treat their pain and were hesitant to refer them to specialist care. Furthermore, adolescents described how being referred to a psychologist or specialist was associated with long waiting times, prolonging their suffering.

Adolescents found it difficult to carry information on previous treatments and the status of their conditions from one HCP to another, as it was hard to remember every detail. This places stress on them as they fear giving incorrect information, and it is exhausting to recount their medical history to every new HCP. Several adolescents expressed the need for enhanced information transfer to improve the efficiency of the referral process.

Adolescent 2: Yes, it’s so true that you forget … Then they ask, yes, what have you been told? When did you have an X-ray or whatever, and it’s like … What did they say you had? And then it’s like … Oh yes, it was some kind of wear and tear on something … And then suddenly it’s something quite minor, and you say it wrong, and then you must start over.

HCPs identified challenges related to disjointed assessment processes. The collaboration between different HCPs and schools was problematic due to a lack of knowledge about each other’s services and a lack of clear guidelines for cooperation in primary healthcare services. HCPs and teachers express that this lack of sufficient collaboration hinders the interdisciplinary teamwork essential for treating complex and multi-faceted conditions. GPs described how they experienced adolescents with long-lasting pain falling between two boxes as they recognized their pain but did not consider it severe enough to necessitate intervention from a psychologist. Thus, GPs often find themselves in a situation where they see a need but lack referral options going forward.

GP: We end up just hitting a wall and waiting for things that never happen. Because these patients are often not sick enough to get into the Child and Adolescent Psychiatric Services. The worst cases get in there, but I think there’s a middle group here that would benefit greatly from psychological treatment, but I can’t help them.

Another common aspect was how adolescents and parents had experienced how teachers were ill equipped to handle adolescents with supportive needs from long-lasting pain, especially if there was no established diagnosis. This led to tensions in adolescents, parents and the teachers’ relationship. Contrarily HCPs described how youths did not always understand how to carry out recommended treatments in everyday settings or explaining them to teachers. All participants agreed that it was important to balance adolescents’ school tasks and ensure they had space to manage their conditions. However, the lack of communication between HCPs and teachers and shared vocabulary for collaborating and implementing treatments in school settings was a barrier.

Adolescent 2: Because at school, for example, they won’t exempt you from the attendance limit or from physical education or such things, unless you have documentation that you have a reason to be exempted. And if your doctor says, no, there’s nothing wrong with you, just take some painkillers and it’ll pass. Then you won’t get that documentation, which will then affect everything else.

Economic factors also emerged as a significant barrier to accessing and receiving quality care. Adolescents reported that the costs associated with GP consultations, necessary for obtaining school absenteeism approval, became a burden leading them to postpone seeking care.

### Theme 6: Visions for improving treatments

Participants shared a variety of visions for improving support for adolescents with long-lasting pain on both individual and systemic levels. An overarching vision of integrated support emerged, emphasizing the importance of collaboration across multiple domains, including peer and parental support, the development of digital tools, and changes in healthcare and educational systems.

#### Peer and parental support

Adolescent participants suggested how a pain camp could provide a safe space to receive patient education, meet other adolescents with pain and share their experiences and learn from each other.

Adolescent 4: Yes, and also to get the opportunity to talk about pain. I mean, Aisha (case 3) found it very difficult to describe what it was like to be in pain. And it was hard to articulate. And when you hear others talk about it, it suddenly becomes much easier in a way.

This suggestion was echoed by the parents who envisioned a support group where parents could share experiences on supporting their child with long-lasting pain and navigating the health system. Adolescents envisioned retreats to warm locations and GPs prescribing nature walks to alleviate stress and promote physical and mental well-being. However, they emphasized that nature walks should be monitored to ensure safety in case of a flare-up when far from help.

#### Digital solutions

Adolescents and HCPs suggested incorporating digital solutions – either as platforms or apps – to support pain management, prevent information loss and helping young people and parents navigate the health system. Adolescents and HCPs envisioned a digital portal, with reliable health information tailored to adolescents like e.g. treatment options, contact to HCPs and specialists and an anonymous chat function to support physical- and psychological self-management, while acting as a hub for multidisciplinary follow-ups on treatments. HCPs envisioned the portal reducing adolescent’s stigma when seeking treatment.

Adolescents and HCPs envisioned having an app where adolescents inserted in their personal information to receive AI tailored exercises, mindfulness and management advice, thus empowering their development of personal management strategies.

Adolescent 7: We talked about an app that was free, of course. It gave you either relaxation exercises, audio files with guided hypnosis, workout plans, diet stuff and so on, which are tailored to each individual with different problems and different pains. A person who gets migraines from running can’t be told that you have to run.

Finally, adolescents suggested how a journal feature could help youths with articulating their pain to GPs and other HCPs during consultations. Still, both HCPs and adolescents mentioned fear of digital solutions replacing face-to-face contact, or adolescents with pain having trouble looking at screens, as barriers for digital solutions.

#### Healthcare system changes

Adolescents envisioned longer consultation times, free and improved access to GPs. This would help them obtain documentation for absenteeism and provide sufficient time to discuss their pain and management challenges. HCPs and adolescents envisioned improving referral options and including time for a thorough, holistic examination to identify possible psychological components to adolescents’ pain.

GP: I’ve yet to see adolescents with long-lasting pain … I’m not talking about three months, but really persistent. That such situations exist without some kind of troubling background. Whether it’s neglect or abuse. There’s usually something there that’s difficult to uncover.

School health nurse: Of course, if you’ve been carrying a worry and a burden for a long time, it manifests in many ways. So, it’s not necessarily wrong. But it’s about how you approach it. Understanding that it could be a psychological issue or an experience you’ve had, something you carry with you.

All groups described that it was difficult for adolescents and parents to keep track of treatment options and carrying information between HCPs. Adolescents, parents, HCP and teachers envisioned incorporating a health coordinator who follows and helps adolescents and parents navigating the healthcare system and communicating with different HCPs to ensure continuity of care, and effective information exchanges during referrals.

Alternatively, adolescents, parents and HCPs expressed how referring adolescents to a multidisciplinary team could have similar benefits, as such team could provide a thorough examination, ensuring the bio-psycho-social aspects of adolescents’ situations were discussed, making treatment plans, promoting HCP collaboration while removing adolescents’ needs for carrying information between HCPs. Furthermore, the multidisciplinary team should also have contact with teachers and school health services to ensure treatments were integrated into school settings.

Adolescent 1: I feel that they, like doctors, psychologists, neurologists, need to improve their communication. Because one can get very tired of going back and forth. And then you have to tell the same story over and over again. And they need to get better at actually communicating.

Furthermore, adolescents suggested creating a GP who specialized in adolescent illnesses, understood their life situations and was trained in communicating with adolescents, parents and teachers to ensure that adolescents didn’t feel overlooked or embarrassed when seeking care.

Adolescent 5: It makes him (case 2), feel embarrassed to go to the doctor. He doesn’t know what he will be met with. But if there is someone who automatically thinks that: yes, of course, even if you are 15 or 20 years old and have complaints (..) I will of course take you seriously (..). If you are met by someone who knows how to meet you, it becomes a bit safer.

#### The educational and school health services

All participants agreed on the importance of balancing the adolescents’ workloads and strengthening education and health system collaboration, to avoid overburdening adolescents. HCPs, adolescents, and teachers envisioned making it easier for adolescents to obtain tailored education activities. This included removing grades to reduce stress, allowing adolescents to withdraw if needed, and providing access to special tools such as elevator cards for easier mobility. Adolescents highlighted how this required adolescent-teacher trust to be fruitful.

Adolescent 6: I feel there is very little trust between students and teachers. I feel teachers need to have more understanding that not everyone is trying to skip school, and not everyone is trying to bypass assessments. I have friends and have experienced having pain and injuries but still trying to attend the most important classes. And then one should not be knocked down and drop out of school because one physically cannot make it to the other classes.

Furthermore, parents and adolescents suggested incorporating patient education classes or support groups during physical education classes which adolescents with chronic pain often are exempt from. These classes could include physiotherapists, school nurses and teachers to educate adolescents in self-management, exercises, career advice and help to navigate educational support options. This could also help adolescents feel they are doing something proactive and reduce stress.

Parent: So that perhaps instead of having regular physical education classes, one could come and have a plan with the physiotherapist. So that one gets a feeling that what I’m doing, it’s for my health, and it’s something that I know is good.

Adolescents, parents and HCPs all suggested strengthening the mandate of the school health services and including them as a resource in treatments. Adolescents envisioned how adolescents could consult the school health services in case of absenteeism rather than having to spend time and money on consulting their GP. Furthermore, adolescents and GPs suggested that the school health services could help implement patient education, coordinate between HCPs and teachers, and educate teachers and ensure treatments were worked into education settings to support adolescents’ in mastering their conditions.

Finally, all participant groups suggested that the school health services could have a preventative role by scheduling yearly health check-ups for adolescents to identify the ones who hadn’t sought treatment for long-lasting pain, to ensure they received adequate support.

### Matrix analysis

**Fig. 3:**
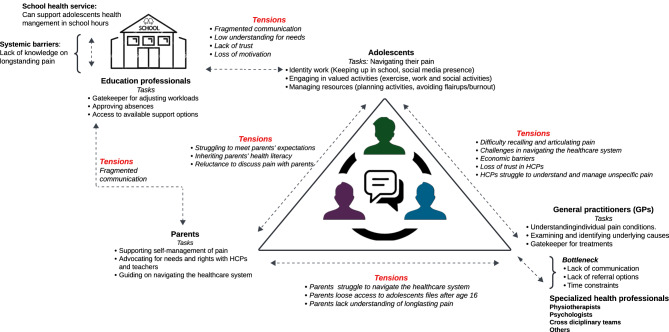
A conceptual model illustrating tasks and tensions. *This conceptual model outlines the various* **domains**, *actors, roles, and tensions present within the design space. The domains represent key areas of focus for intervention, such as healthcare settings, educational environments, and family dynamics.* **Actors** *include adolescents, parents, healthcare professionals, and educators, each playing distinct* **roles** *in the management and support of adolescent pain. The model highlights existing* **tensions**, *such as communication barriers and systemic barriers like limited time and resources*

The matrix analysis synthesized the themes from each workshop to create a conceptual model illustrating the domains, actors, roles, interactions and tension points as possible targets for future interventions for improving adolescent’s individual and shared- management of long-lasting pain (Fig. 3). Adolescents occupied a central position within the model, interacting with parents, peers, HCPs, and teachers, navigating tasks like understanding their pain, identity work and avoiding stigma. GPs inhabited an adjacent domain as gatekeepers; tasked with understanding adolescents’ individual conditions, diagnosing- and providing treatment access, while parents took a mediating role which included providing adolescents with management support, psychological support, negotiating with HCPs and teachers and helping adolescents navigate the health system. Teachers’ roles included supporting adolescents’ academic performance, adjusting adolescents school workloads, approving absences and access to school health services.

Our conceptual model identified several types of barriers which impaired task performance, collaboration and caused tensions within the collaborative space. The most prominent barrier related to the invisible nature of long-lasting pain, causing stigma which impeded adolescents’ performance and ability to express their pain and ask for help. In the adolescent-HCP relationship, tension sources included adolescents with long-lasting pain often struggling to recall and articulate their pain, which hindered HCPs’ understanding of their conditions and life situations and affected their ability to find appropriate pain management solutions. Tensions within the adolescent-parent relationship emerged from parents’ lack of knowledge of pain, and the real or perceived expectations for the adolescent which complicated communication. Furthermore, loss of access to adolescents’ files and lack of understanding of the healthcare system caused tensions and prevented parents from assuming the roles as supporters and advocates for the adolescents. Finally, the gap between adolescents, parents, HCPs and the education- and school health professionals caused tension due to fragmented communication, and the teachers limited understanding of long-lasting pain, which inhibited collaboration and adolescents’ ability to access support from the school health service during school. By taking point in the identified visions, the tension points can be utilized to inform future intervention designs to improve communication and encourage collaborative care.

## Discussion

### Key findings

This study explored the lived experiences and visions of adolescents with long-lasting pain, parents, HCPs and teachers for a person-centered intervention. Participants highlighted the impact of pain on adolescents’ lives, their daily-wellbeing, academic performance, social interactions and identity. Furthermore, adolescents reported management challenges emerging from a lack of recognition from others, fragmented communication and collaboration and systemic barriers.

A key finding was the complex interplay between adolescents’ efforts to manage pain, and parents, HCPs, and teachers’ supportive roles in self-management, highlighting needs for better communication to build collaboration and trust. Adolescents struggled with navigating pain management, social obligations, and learning to maintain an equilibrium through trial-and-error. Parents acted as coordinators, emotional supporters and navigators of school and healthcare systems. HCPs challenges included balancing diagnosing, providing treatments, validating the adolescents’ experiences and navigating time constraints. Teachers were responsible for adjusting adolescents’ work burdens, making space and providing access to school-based support.

The matrix analysis identified individual and systemic communication barriers as sources of tension and design targets for patient-centered interventions. Adolescents described how having time and space to manage pain, along with support from parents, HCPs, and teachers, aided their self-management efforts. Schools emerged as intervention targets, with envisioned features including early holistic assessments, interdisciplinary teams, HCPs specialized in adolescents, digital tools and enhanced school health services. These features should focus on pain management, while strengthening stakeholder communication to create supportive environments and reduce actors’ management burden.

### Adolescents’ challenges with pain management

Adolescents experienced their pain as dynamic, limiting their participation in education-, work- and social activities and affecting psychological wellbeing, as shown in similar studies with adolescents with long-lasting pain [8, 29, 33, 34]. Adolescents described struggling to maintain normalcy with their pain as described by Spencer et al. [61]. Thus, their management became an ongoing effort with balancing their pain, social demands and maintaining a certain identity, aligning with Corbin and Strauss [62] three lines of illness management. Our analysis identified how adolescents developed personal management strategies via a trial-and-error approach, like Lorig and Holman [63] documented in adults. Furthermore, adolescents initially reported pain-related worry and fear, but this dissipated over time, suggesting that self-efficacy may have played a role in mitigating adverse pain beliefs [13]. Thus, improving adolescent’s self-efficacy through patient education, exercises, and self-guided trail-and-error should be explored in future interventions [63, 64]. Our analysis described how adolescents’ self-management had a social dimension, as their efforts towards keeping up with friends, social activities and schoolwork could lead them into a ‘boom and bust’ cycle as described by Kempert [65] with high activity and forced activity withdrawal. Adolescents experienced that expressing their pain verbally or withdrawing exposed them to stigma [66], making them reliant on parents or teachers recognizing their pain to obtain management support. While qualitative studies highlight how living with pain can impact adolescents’ identity development [14, 26], adolescent participants described sacrificing activities to maintain a certain identity (e.g. the good student). Failing to make this transaction could lead to demotivation and gradually increase activity avoidance, as described in adults with chronic pain [67]. Contrarily, cultivating trusting working alliances with parents, HCPs and teachers was described as important to ensure adolescents had the space and support needed to facilitate their transition into self-management [68].

### Roles, tensions and barriers

Our analysis identified a complex relationship of interactions between adolescents with long-lasting pain, parents and HCPs as described by Brooker et al. [69] and Johansen et al. [40]. Our study expanded upon these by outlining the education system as an arena where adolescents experienced pain, performance demands and management needs [17, 61].

#### Roles and expertise of parents, HCPs and teachers

Other stakeholders occupied different, dual roles within the complex care setting; using their expertise to develop adolescents’ health literacy and self-management competencies as exemplified by Cha et al. [70]. Parents of adolescents with long-lasting pain often assume informal carer responsibilities [71], and our findings illustrated how parents alternated between supporting their child and advocating with education- and health professionals. HCPs faced dual responsibilities as suggested by Engebretsen [72], using their clinical expertise for diagnosis and treatment, while engaging in patient education and referral decisions when needed. Similarly, teachers’ roles mirrored observations from two reviews [61, 73] as education professionals acted as gatekeepers for academic accommodations, access to support and absenteeism, while using their expertise to negotiate with parents and adolescents to reach education goals.

#### Communicative and systemic barriers

Several barriers contributed to tensions within the care continuum. While GPs played a crucial role in helping adolescents understand their pain [33, 74, 75], adolescents’ challenges with articulating their pain and the limited health literacy of both parents and adolescents complicated clinical interactions, as documented by Brown et al. [31]. Additionally, adolescents and parents often struggle to adjust to the condition and diagnostic uncertainty [76]. Our analysis showed that not getting an answer or tangible solution to pain complaints reinforced mistrust and stigma, as documented in previous studies [74, 77, 78]. While qualitative studies highlight how a non-judgmental approach, patient involvement and trust are key for patient buy-in [79, 80], our data confirmed this as participants highlighted how transparency and inclusivity in HCPs decision-making fostered trust and reduced tensions. Parental support has been linked to improved pain acceptance, management and quality of life in youths with chronic pain [81, 82]. However, parental inflexibility and adolescents’ reluctance to self-disclose their pain was a source of tension, inhibiting the role negotiation Leonard et al. [83] highlights as essential for shared decision-making.

Systemic barriers contributed substantially to tensions. While cognitive behavioral therapies (CBT) have demonstrated improved outcomes in managing long-lasting pain in adolescents [24, 84, 85], limited access to psychological support and waiting times added to tensions. While teacher inflexibility and dismissal constitutes a major barrier for adolescents’ management of chronic illness [17, 86], participants highlighted how school accommodation and work reductions often required a medical diagnosis, acting as a barrier for adolescents’ receiving management support during school. Our results, along with patient studies, show that poor communication often forces adolescents and parents to relay health information between actors, increasing risks of information loss and fragmented care [87]. Finally, long-lasting pain is associated with education anxiety, reduced academic functioning and challenges with accessing support [16, 17, 88]. Our findings suggest that improving communication between HCPs and the education system may strengthen support networks for adolescents with long-lasting pain.

### Visions for future interventions

Adolescents with long-lasting pain often receive inadequate care [89, 90]. Participants envisioned healthcare system changes that could improve adolescents’ access to quality care, suggesting the inclusion of a healthcare coordinator, referrals to multidisciplinary teams, and allocating more time for consultations. Studies on interdisciplinary pain treatment demonstrate significant improvements in pain intensity, disability, and school attendance, along with moderate benefits for anxiety and depression [91, 92]. These positive effects are attributed to HCPs collaborating to address the bio-psycho-social aspects of adolescents’ pain experiences, thereby offering integrated and comprehensive care and support [93]. Further, adolescents in our study envisioned the inclusion of GPs specialized in adolescent health to improve patient-HCP communication. Providing GPs with training in adolescent medicine has been shown to increase their confidence in discussing difficult topics during consultations [94].

#### Digital solutions

Participants envisioned using digital tools and platforms to support adolescents’ individual management decisions, while creating digital pathways for enhancing HCP-communication and access to education resources. Digital interventions have shown promise in enhancing pain management, disease understanding, psychological well-being and self-efficacy among adolescent patients [95–97]. Furthermore, a digital platform could help alleviate problems with disjointed communication, allowing patients to distribute communication with several HCPs [98]. A content analysis identified a range of digital health interventions, including apps [99], and several online platforms (e.g PainBytes, Comfortability, ImaginAction) exist with resources for providing reassurance, supporting pain reduction, and delivering management education for adolescents with long-lasting pain. However, adolescents’ challenges with identifying quality digital resources, language barriers and their struggles with screen usage and concentration [28, 100], makes it crucial to understand how to design and deliver digital patient information in a way that is identifiable, actionable and supports collaborative care.

#### School-based interventions

Schools were identified as promising arenas for interventions. School-based interventions, including exercises and self-management education, have shown positive effects on adolescent pain outcomes [20, 27, 101, 102]. Participants recommended that school health departments play a mediating role in managing adolescent pain. Research suggests that school nurses are well-positioned to identify and educate students experiencing long-lasting pain [103]. These school health personnel could serve as contact points for multidisciplinary teams, facilitating the implementation of interventions and addressing teacher inflexibility, which is a major obstacle in educational settings [61]. However, achieving this might require additional resources, such as increasing the number of school nurses.

### Strengths and limitations

A key strength was our inclusion of diverse stakeholders – adolescents, parents, HCPs, and teachers – allowing for a holistic understanding of pain management across contexts. While 11 participants were included in workshop three, only four parents attended, resulting in the workshop didn’t meet the recommended 8–16 participants [58]). This was a limitation as parents’ perspectives may be disproportionately represented within the analysis. The use of inspiration cards [49, 50] and case vignettes [48] facilitated meaningful discussions, and has been successfully implemented in previous research [40, 104]. Nonetheless, the workshop methods’ reliance on participants’ recalled experiences introduces potential recall bias [105]. Efforts were made to mitigate this through structured discussions and case-based prompts. Also, the data on diagnoses were self-reported and not clinically verified, which may have introduced variability in how participants interpreted the question or reported their diagnoses. Additionally, all adolescent participants had sought medical help, which may have influenced their perspectives, a factor to consider when designing future interventions for adolescents who have not yet engaged with health services.

### Future research directions

Based on our findings, future research should explore how interventions for adolescent pain management can be implemented in real-world settings and influenced by contextual factors such as schools, families, and healthcare systems. This includes understanding how these factors shape the uptake and effectiveness of interventions and how to best support collaboration between stakeholders across multiple domains. Investigating ways to adapt interventions to adolescents’ diverse and changing information and support needs and everyday environments may provide valuable insights for improving outcomes. Although not raised by participants in this study, future research should also examine the role of sleep hygiene and its integration into self-management strategies for adolescent pain.

## Conclusion

This study identified barriers and proposed intervention strategies to enhance care for adolescents with long-lasting pain. Communication challenges between adolescents, parents, HCPs, and teachers contribute to trust issues and inhibited collaborative care, while systemic factors such as time constraints and limited treatment options exacerbate these difficulties. Participants envisioned early holistic assessments, designated care coordinators, and the use of digital platforms to improve communication. Schools were identified as viable settings for interventions. The involvement of end-users in this study can help align future interventions with their actual challenges and needs, thereby increasing their potential impact.

## Electronic supplementary material

Below is the link to the electronic supplementary material.

Supplementary Material 1

Supplementary Material 2

Supplementary Material 3

Supplementary Material 4

## Data Availability

The data supporting the findings of this study are not openly available due to sensitivity concerns and privacy regulations. Data sharing can only occur after the deletion of recordings and any identifying links. The data are securely stored in controlled access data storage at TSD (Tjenester for Sensitive Data), managed by the University of Oslo.
