# Supplementary material for: Co-creating support for adolescents with long-lasting pain: findings from workshops with adolescents, parents, and professionals
Source: BMC Health Serv Res. 2025 Nov 24;25:1516. doi: 10.1186/s12913-025-13654-0 (PMC12645688; doi:10.1186/s12913-025-13654-0)
Supplement: Supplementary file 1 — Supplementary Material 1 [file 12913_2025_13654_MOESM1_ESM.docx]

**Appendix 1.**

**Case-vignettes for the workshops**

1. Sofie is 16 years old and has just started her general studies specialization at a high school on the west side of Oslo. She lives with her mother and her 12-year-old brother and stays with her father every other weekend. Her mother is a lawyer and works a lot. Sofie is very ambitious at school and receives good grades, but she finds it stressful and struggles to find time for everything she needs to do. She often stays up late at night with homework. Her goal is to get into medical school in Norway after high school. She is very social, has many friends, and regularly goes to the gym. She is active on social media and has more than 2,000 followers on Instagram.

Sofie began experiencing neck pain after a couple of months in high school. The pain developed gradually, and she doesn't know the cause. She feels the pain daily, and it gets worse in the evening, often leading to headaches. She takes over-the-counter painkillers 3-4 times a week. Taking painkillers helps with the worst pain, but she still feels a mild pain in her neck and head that doesn't go away. The neck pain makes it challenging to do schoolwork, and she is worried it will affect her grades. She is also concerned that the neck pain might be something serious and won't go away. She is now at the point where she needs help but is unsure where to turn.

2. Lars is 17 years old and is in his second year of carpentry training. He lives with his mother, who is disabled, and his father, who is a carpenter. He has always found school challenging and was unsure about what to study after junior high. He chose carpentry because he finds theoretical subjects difficult and felt he needed to start a vocational education. He is nearly finished with his second year and is thinking about where to apply for an apprenticeship for the next two years. In his spare time, he tries to relax as much as possible to be ready for physical work at school. He doesn't feel the need to exercise because he is already very physically active at school. He enjoys gaming and socializing with friends in this way.

He has had quite a bit of absence from school due to pain in both shoulders. He believes it is caused by the heavy practical work involving his arms. The pain often occurs while he is working and worsens throughout the day. He has to take frequent breaks and feels that his classmates see him as lazy because of this. The shoulder issues make it challenging for him to choose an apprenticeship, as he is unsure whether he will be able to handle such a physically demanding job. He is frustrated because he doesn't see any other solution for what to do. He receives little understanding from his father, who is in the same profession, while his mother is more understanding as she struggles with similar issues. He has not sought help for his problems, as he feels embarrassed, and his father thinks he should just tough it out. His mother, however, wants him to go to his general practitioner to get professional help.

3. Aisha is a 17-year-old girl in her second year of high school who comes from a minority background and lives on the outskirts of Oslo with her parents and four siblings. Over the past year, Aisha has experienced recurring and unclear pain in her knees and hips, and sometimes in her back and neck. She has also struggled with overweight issues for a long time.

Aisha tries to be mindful of her diet and has received dietary guidance, but often feels powerless because her family's eating habits are difficult to change. She also started going to a gym with a personal trainer twice a week. However, Aisha experienced increased pain and discomfort during workouts and dreaded each training session. She felt that the trainer didn't take her needs and pain condition into account, which made her fear that exercise could worsen her situation. Aisha is now uncertain about what her body can actually handle in terms of physical activity. As a result, she has gradually become more inactive in recent months, and the pain has become more frequent and persistent. Aisha has sometimes avoided going to school on days when the pain feels intense. She has also started taking pain medication, initially occasionally, but now several days a week.

Aisha has parents with a caring but sometimes demanding parenting style. For several years, she has worked some days after school, but due to her pain condition, her parents have now recommended that she take a break from work. They are concerned about her health and encourage her to see her general practitioner for help. Although Aisha is open to seeking help, she finds it challenging to explain how the pain occurs and feels down and frustrated when trying to convey her situation to others. She also finds it difficult to open up and share her feelings, especially with her parents.
