# Supplementary material for: Co-creating support for adolescents with long-lasting pain: findings from workshops with adolescents, parents, and professionals
Source: BMC Health Serv Res. 2025 Nov 24;25:1516. doi: 10.1186/s12913-025-13654-0 (PMC12645688; doi:10.1186/s12913-025-13654-0)
Supplement: Supplementary file 2 — Supplementary Material 2 [file 12913_2025_13654_MOESM2_ESM.docx]

| **Appendix 2.**  **Card game for Workshops** | |
| --- | --- |
| **Type of cards** | **Description** |
| **Domain cards:**  Physical domains - where adolescents experience pain | At school    At home  During leisure time   - With friends - During sports participation   At healthcare providers  Blank cards |
| **Problem cards:**  Problems or challenges | The pain experience    Communication  Low health literacy  Understanding of pain   - Fear of pain (serious injury/condition) - Theories and beliefs about the pain - Pain triggers and pain relievers   Motivation  Information from external sources, Dr. Google?  Consequences for participation   - School - Friends - Activities   Isolation  Mental health challenges   - Identity, future dreams - Hopelessness, frustration, energy level, tiredness, psychological distress   Blank cards |
| **Solution cards:**  Place possible solutions alongside the problem card + domain cards | School-healthcare     - School nurse - School physiotherapist   School system  General practitioners  Physiotherapist -examination and follow-up  Help from a multidisciplinary team  Examination  Pain neuroscience education – information on what causes pain   - Recognizing patterns of pain - Learn about load management (total workload)   Psychological therapies (Cognitive Behavioral Therapy)  Relaxation techniques (help to relax - for instance Yoga, mindfulness, breathing exercises)  Exercise instructions or activity plans  Patient stories or support groups, description of adolescents with pain and how they manage their pain condition  Digital solutions (apps, website)  A logbook or a diary for tracking their pain  Co-developing an action plan with healthcare providers  Thorough examination  Nature exposure  Blank cards |
| **Examples from group works** | 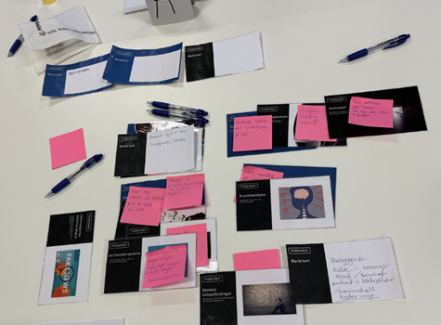  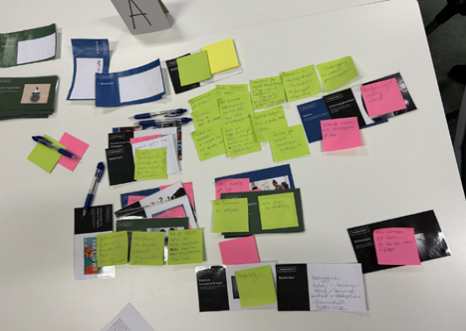 |

**Procedure and use of cards in the workshop phases**

| **Phase** | **Procedure and use of cards** |
| --- | --- |
| Critique phase | Participants used Domain and Problem cards to identify and discuss challenges across different contexts. |
| Fantasy phase | Participants brainstormed solutions by adding Solution cards to the identified challenges. |
| Implementation phase | Participants refined and prioritized ideas from the Fantasy phase using Solution cards, focusing on actionable steps. |
