# Supplementary material for: Co-creating support for adolescents with long-lasting pain: findings from workshops with adolescents, parents, and professionals
Source: BMC Health Serv Res. 2025 Nov 24;25:1516. doi: 10.1186/s12913-025-13654-0 (PMC12645688; doi:10.1186/s12913-025-13654-0)
Supplement: Supplementary file 3 — Supplementary Material 3 [file 12913_2025_13654_MOESM3_ESM.docx]

| **Appendix 3.**  **Finalized Themes Identified in Workshops (Step 5 of Braun & Clarke's Reflexive Thematic Analysis)** | | |
| --- | --- | --- |
| **Workshop 1: Adolescents** | **Workshop 2: Healthcare personnel and teachers** | **Workshop 3: Parents** |
| **The pain experience**  - Challenges in expressing pain and feelings of isolation.  - Invisibility: Pain often unseen, fluctuates, and impairs activities.  - Articulating pain: difficult to describe and fear of misunderstanding; mental impact harder to express.  - Stress and anxiety about worsening condition, misdiagnosis, and physical decline. | **Adolescents’ challenges in expressing and communicating pain**  - The pain may have a function → acceptance of limitations.  - Shame may prevent seeking help. | **Challenges navigating the healthcare system**  - Unclear pathways for seeking help and follow-up after tests.  - Loss of parental access to health information after age 16.  - Navigating the system demands large efforts from youth and parents. |
| **Impact on mental health**  - Pain disrupts school, social activities, and overall well-being.  - Struggles with school and lack of understanding cause stress.  - Lack of progress leads to frustration.  - Social limitations may lead to isolation.  - Constant struggles with pain create a sense of hopelessness.  - Pain reduces motivation and self-esteem, affecting school and social life. | **Performance pressure as a cause and/or sustaining factor of pain**  - High expectations and pressure to achieve career goals.  - High stress levels → intensifies the pain experience.  - Reduce performance pressure to prevent stress-related pain. | **Inadequate Recognition of Pain**  - Pain often minimized: a lack of empathy and trivialization from school and healthcare.  - Frustration over no clear diagnosis  - Negative impact on mental health and academics. |
| **Pain management strategies**  - Modify or avoid activities to balance pain, risking being seen as lazy.  - Resource Management: Prioritize tasks and plan to prevent burnout.  -Skip social activities to minimize pain → frustration and feelings of failure.  - Ignore or hide pain to avoid stigma.  - Recognize the role of nutrition  - Frequent painkiller use, often unaware of long-term risks. | **Pain's impact on motivation in school, activities, and social life**  - Low self-confidence and perception of others’ greater abilities → increased focus on pain.  - Activity cessation and isolation.  - Cycle of hopelessness.  - Fear of worsening condition. | **Insufficient knowledge among GPs**  - Limited understanding of non-specific symptoms.  - Parent frustration: significant effort required to gain knowledge.  - Information challenges: difficulty for parents to access and understand relevant information. |
| **Not feeling understood**  - Lack of support from peers and teachers leads to feelings of isolation.  - Adolescents often feel dismissed by healthcare providers, causing uncertainty and stress.  - Inadequate parental understanding diminishes motivation to seek help. | **Health literacy and cultural understanding among minority adolescents**  - Lack of understanding of the Norwegian healthcare system  - Language barriers.  - Cultural differences: mental health issues are more stigmatized.  - Increased cultural competence needed for healthcare professionals. | **Challenges of Parenting Adolescents with Non-Specific Pain**  - Communication issues: Adolescents ignore parental advice.  - Parents must manage care and take a coordination role.  - Parents need support. |
| **Economic factors when seeking care**  - Fees for GP consultations hinder access to necessary care and absenteeism approval.  - Multiple consultations and specialist referrals increase financial burden, discouraging care-seeking.  - Loss of Autonomy: Dependence on parental financial support reduces adolescents' sense of independence. | **Incoherent evaluations and challenges in identifying explanatory models**  - GPs are unsure of school health services offer for pain management.  - Dilemma of time constraints vs. need for imaging diagnostics.  - Integration of a biopsychosocial understanding of pain. | **Challenges related to school absenteeism**  - Non-specific pain: Difficulty obtaining valid documentation.  - Adolescents face stress from academic pressure.  - Struggles with performance lead to decreased motivation.  - Vicious cycle: Pain, absenteeism, stress, isolation, and low mood. |
| **Exercise and keeping active**  - Recognize benefits of exercise (better mood, sleep, health) but struggle to avoid exacerbating pain.  - Pain Dilemma: Overdoing exercise can cause pain and reduce energy, limiting other activities.  - Planning Required to balance exercise with pain management and social activities → Support Needs: knowledgeable trainers and supportive schools to manage exercise and pain. | **Meaningful activities and coping**  - Engagement in meaningful activities.  - Enhances quality of life.  - Concern/barrier due to high costs. | **Insufficient resources in schools and healthcare**  - Not enough school health nurses to support students.  - Long wait times for examinations and treatment due to general resource shortages. |
| **Adolescents’ roles**  - Multiple roles: Adolescents balanced pain management with school, work, and activities.  - Impact of persistent pain: disrupted routines and free time, and affected school, tasks, and social life. | **Parental support and coping**  - Distraction and minimization vs. over-focus on pain condition.  - Low health literacy → counterproductive advice. | **Financial challenges**  - Expensive exercise options limit access despite their benefits for pain management.  - Cost of valid documentation: Expenses for obtaining necessary documentation can result in unexcused school absences. |
| **Parents roles**  - Parents impact pain management through support and communication.  - Their knowledge of pain affects adolescents' care-seeking.  - Can enhance pain management by sharing insights, offering emotional support, and guiding health decisions. | **A trusting relationship with healthcare professionals**  - Create a safe environment.  - Conduct comprehensive assessments.  - Challenges related to limited time. | **Challenges with social participation**  - Social isolation: Adolescents with pain often withdraw from activities.  - Limited energy spent on school leaves little for social engagement.  - Reduced social interactions may lead to higher awareness of pain. |
| **HCPs roles and responsibilities**  - School nurses are key for support and accommodations, but their limited availability calls for more staff.  - GPs as gatekeepers: mixed experiences.  - Psychologists essential (limited access).  - Physiotherapists are helpful but hindered by delays and poor follow-up.  - Specialized clinics: hard to access due to delays, referral hurdles, and costs.  - Poor communication and fragmented care complicate treatment. |  |  |
| **Education professionals’ roles**  - Teachers are key in supporting pain management but often lack understanding and knowledge about chronic pain → hinder support.  - School health staff face limitations due to systemic barriers. |  |  |
| **Cross disciplinary teams**  - Benefits: Positive experiences with teams due to diverse perspectives, improved communication, and reduced need for repeating information.  - Challenges include lack of adolescent-specific teams and issues with limited benefits or continuity of care. |  |  |
| **Friendships**  - Persistent pain complicates maintaining friendships and social status due to pain management and activity limitations.  - Affects ability to keep up with school, sports, and social media, potentially reducing social reach and engagement.  - Pain and fatigue make it hard to participate in social and leisure activities, risking social isolation and impacting self-esteem. |  |  |
| **Academic impact of persistent pain**  - Decreased ability to maintain academic performance.  - Increased effort required for studying and preparation, with low grades impacting motivation.  - Pain affects concentration during tests.  - Difficulty obtaining support from teachers and health professionals, often requiring formal diagnoses. |  |  |
| **Communicating with HCPs**  - HCPs may lack specific competence for their pain conditions.  - Difficulties include repeating histories, short consultation times, and ineffective communication.  - Pain is not taken seriously.  - Conflicting information.  - Challenges with no/unclear diagnosis. |  |  |
| **Managing school**  -Strategies; building teacher trust, using school resources and special privileges.  - Supportive teachers; trust and understanding are key.  - Special privileges hard to obtain without proper documentation. |  |  |
| **Gender differences in management**  - Males might downplay pain, while females may be more vocal but not always taken seriously.  - Societal and family norms impact these differences. |  |  |
| **Visions:**   - **Future Treatment Programs:**   - *Pain Camps/Support Groups*: share experiences, receive peer support, and gain new pain management strategies.  *- Trips Abroad:* trips to warm climates or specialized centers.  *- Nature Walks:* Incorporating nature walks into treatment plans.   - **Digital Health Solutions:**   *- Health Portal:* A platform for direct communication with healthcare providers, uploading health data, and managing treatments.  *- mHealth App:* An app offering educational resources, treatment tracking, and tailored exercises, with features like AI for personalization and a pain journal to track progress.  *- AI Features:* Use of AI, including text-to-speech options, for adaptive exercises and minimizing strain from device use.   - **Healthcare System Changes:**   *- Easier GP Access:* Online consultations and longer appointment times to improve care and patient experience.  *- Simplified Referrals:* Streamlining referrals to specialists and removing costs for GP visits to reduce barriers for adolescents.  *- Multidisciplinary teams* to provide comprehensive care and reduce the need for adolescents to transfer information between providers.  *- Specialized Adolescent HCPs* **→** to better meet and address unique needs of this age group.   - **Educational and School Health System Enhancements:**   *- Flexible Education & Support:* Adapt schedules and requirements, including removing grades or providing privileges.  - *Integrate pain management education* into the curriculum.  - *Establish effective communication* between schools and healthcare providers.  - *Enhance school health services with first-line support*, including physiotherapy and absence approvals.  - *Implement regular health checks* by school health workers.  - Offer dedicated academic support. | **Visions:**   - **Need for thorough assessment and a holistic approach**   - Assessing both psychological and physical aspects of pain.  - Tailor treatment to individual needs.  - Identifying past trauma is crucial.   - **Structured action plans** - Care pathways activated when interventions are ineffective. - Individual plans. - **Coordinator for interdisciplinary care and adolescent specialists**   - Monitor utilized resources.  - Follow-up; treatment plans and aims.  - Network of specialists in adolescent healthcare.   - **A youth-focused health app/website for pain and health education**   - Adolescent-oriented, reliable, self-management skills.  - Challenges with digital tools: digital dependency, reliability, need for human interaction.   - **Enhanced holistic treatment through interdisciplinary collaboration**   - Coordinated collaboration for tailored solutions and closer follow-up.   - **School health services as an important and accessible health resource**   - Ideal setting to initiate healthcare.  - Increased psychological support.  - School health services like occupational health services.  - Enhance teachers' knowledge of persistent pain.  - Screening tools and traffic light models for identifying health care needs.  - Importance of support and adjustments in school.  - Emphasis on preventive measures. | **Visions:**   - **Healthcare Navigation Support**   - Coordinator who provides oversight on contacts and tracks medical visits and findings.  - Physical or digital solution.  - Especially valuable for those without support at home.   - **Empower and Support Parents**   - Provide parents with tools/support to navigate the healthcare system.  -Emotional Support: Create support groups for parents facing similar challenges.   - **The school as an arena for prevention and treatment**   - School-based interventions recommended: Students are less likely to seek help outside school.  - Easy access to help: Provide low-threshold conversations and support.  - Integrate relaxation and support groups into school hours.  - Teach prevention: Include preventive activities in the curriculum.   - **Interdisciplinary team (at school)**   - Enhance school health services: add nurses, psychologists, and physiotherapists.  - Form a collaborative team to develop plans and support.  - Use interdisciplinary approaches to understand and manage pain and related psychological issues.  - Adapt teaching to help students succeed and feel accomplished.  - Proactively reach out to assist those who don’t seek help and/or with limited home support.  - Reduce stigma by making it easier and less stigmatizing to seek help. |
