# Supplementary material for: Co-creating support for adolescents with long-lasting pain: findings from workshops with adolescents, parents, and professionals
Source: BMC Health Serv Res. 2025 Nov 24;25:1516. doi: 10.1186/s12913-025-13654-0 (PMC12645688; doi:10.1186/s12913-025-13654-0)
Supplement: Supplementary file 4 — Supplementary Material 4 [file 12913_2025_13654_MOESM4_ESM.docx]

**Appendix 4. Steps of the thematic analysis process**

| ***ANALYTIC STEP*** | ***PREPARATION*** | ***STEP 1: Familiarization*** | ***STEP 2: Coding*** | ***STEP 3: Identifying preliminary themes*** | ***STEP 4: Theme development and review*** | ***STEP 5: Refinement*** | ***STEP 6: Synthesis*** |
| --- | --- | --- | --- | --- | --- | --- | --- |
| ***Process:*** | Raw data preparation. | Read-through to become familiar with the text; initial ideas and themes were noted down. | Identification and organization of meaning units into codes. | Codes and subcodes were sorted into potential themes. Mind maps and coding were updated. | Themes and subthemes were reviewed and condensed into written descriptions. | Themes were condensed and summarized, overlaps eliminated, and categories regrouped to refine the thematic structure. | Storybook themes were identified and synthesized. A matrix analysis was used to organize themes and identify tensions and divergences across workshop groups. |
| ***Performed by:*** | Audio recordings transcribed using Autotext * service (Whisper) and manually checked by HJ and MHG. | Distribution of analysis-work:   - Workshop (WS) 1 (Adolescents): SKJ - WS 2 (HCPs): MHG - WS 3 (Parents): HJ | Identification and coding of elements within each dataset (Workshops 1, 2, 3; Groups A, B, and C) were conducted by MHG, SKJ, and HJ. A coding list was created for each dataset. | Emerging themes and relationships were discussed among the researchers (MHG, SKJ, HJ) to ensure that the derived themes were logically grounded in the data. | Themes were reviewed and selected collaboratively by SKJ, MHG and HJ. | The refinement, definition, and naming of themes were conducted collaboratively by SKJ, MHG and HJ. | The synthesis and matrix analysis were collaboratively conducted by SKJ, MHG and HJ. |
| ***Tools:*** | Whisper speech recognition system * | By hand. | Nvivo 1.7 (v20) **: mind map and code list. | Nvivo 1.7 (v20) **: mind map and code list. | Word ***: tables for each workshop group. | Word ***: a combined table for all workshop groups. | Word ***: story-books compiled into a text forming the results.  LUCIDcharts ****: Fig.3 (conceptual model). |
| ***Units of analysis:*** |  |  | WS1: 7 supertype themes, 44 themes, 41 sub-themes; WS2: 16 supertype themes, 31 themes, 25 sub-themes; WS3: 11 supertype themes, 49 themes, 9 sub-themes. |  | WS1: 17 themes, WS2: 14 themes, WS3: 11 themes | WS1: 17 themes, WS2: 9 themes, WS3: 9 themes (Appendix 3) | 6 storybook themes and a conceptual model (Figure 3). |
| ***Evaluation:*** |  |  | Testing of coding by co-authors TBS and KR. |  |  | Themes were reviewed and validated by co-authors TBS and KR. | Review, input and validation from all co-authors. |

* Whisper speech recognition system, as implemented in the University of Oslo’s internal transcription service Autotext.

** Nvivo 1.7 (v20) (QSR International Pty Ltd, Melbourne, Australia).

*** Microsoft Word for Office 365 MSO (Microsoft Corporation, Redmond, WA, USA).

**** LUCIDcharts (Lucid Software Inc., South Jordan, UT, USA).

Initials refer to the authors as listed in the manuscript.
